# Comparison of Laboratory Diagnosis of Urinary Tract Infections Based on Leukocyte and Bacterial Parameters Using Standardized Microscopic and Flow Cytometry Methods

**DOI:** 10.1155/2022/9555121

**Published:** 2022-05-27

**Authors:** Priskila Christy, Hanna Evelina Sidjabat, Anggia Augustasia Lumban Toruan, Emmanuel Jairaj Moses, Narazah Mohd Yussof, Yessy Puspitasari, Muhammad Robiul Fuadi, Aryati, Ferdy Royland Marpaung

**Affiliations:** ^1^Department of Clinical Pathology, Faculty of Medicine, Universitas Airlangga, Dr. Soetomo Hospital, Surabaya, East Java 60286, Indonesia; ^2^Menzies Health Institute Queensland, Griffith University, Brisbane, Queensland, Australia; ^3^Department of Urology, Gotong Royong Hospital, Surabaya, East Java 60119, Indonesia; ^4^Department of Biomedical Sciences, Advanced Medical and Dental Institute, Universiti Sains Malaysia, George Town, Malaysia

## Abstract

**Background:**

Rapid and reliable tests are essential for the diagnostic laboratory confirmation of urinary tract infections (UTIs). Until now, UTI has been confirmed by the microbiology culture of urine, requiring at least 48-hour turnaround time (TAT), with a standardized microscopic method being widely favored. Automated urine flow cytometry, however, has recently been used to improve the rapid TAT by analyzing the urine sediment. This study therefore aimed to compare the diagnostic value of the Shih-Yung conventional microscopic and urine flow cytometry methods in the detection of leukocyte and bacterial parameters of patients with UTIs in an outpatient clinic.

**Methods:**

A cross-sectional study was conducted on a total of 100 patients. Seventy urine samples were positive for leukocytes and nitrite chemistry, and 30 were negative for both. The measurements of urine leukocytes and bacteria were compared between Sysmex UF-5000 urine flow cytometry and the Shih-Yung method. The diagnostic value was obtained from ROC analysis of urine flow cytometry and the culture.

**Results:**

A leukocyte cutoff value of 87.2/*μ*L had a sensitivity and specificity of 98.33% and 95%, respectively, and 98.33% sensitivity and 75% specificity at a bacterial cutoff of 582.22/*μ*L. Interestingly, our study identified strong and consistent agreement of leukocyte and bacterial parameters between urine flow cytometry and Shih-Yung (*k* = 0.959, *p* < 0.001 and *k* = 0.939, *p* < 0.001, respectively). Furthermore, through analyzing the dominance angle of the scattergram, a strong agreement was obtained with the culture result (*k* = 0.880, *p* < 0.001).

**Conclusions:**

The Shih-Yung method showed consistent agreement with urine flow cytometry for the detection of leukocytes and bacteria. The use of certain cutoffs for bacterial and leukocyte parameters in urine flow cytometry demonstrated very good performance in detecting acquired symptomatic UTIs.

## 1. Introduction

Urinary tract infection (UTI) is an infection in the urinary tract, affecting the urethra, bladder, ureters, and kidneys [1, 2]. It is categorized as symptomatic or asymptomatic. It is among the most common infections, with an incidence of 150 million cases worldwide each year, with the majority of patients being female and the elderly [3]. The mortality rate for UTI is 2.3%, which rises to 26% if complicated with bacteremia or septic shock [4]. Moreover, UTI is the second most common reason for the use of empiric antimicrobial therapy [5]. Data from several tertiary hospitals in Indonesia show continuous reduced antimicrobial susceptibility patterns and the rise of multiple antimicrobial-resistant bacteria prevalence, such as methicillin-resistant *Staphylococcus aureus* (MRSA) and the use of extended-spectrum beta-lactamase (ESBL) [6]. Approximately 87% of UTIs in Surabaya, Indonesia, are caused by *Escherichia coli* [6]. In terms of diagnosis, UTI is strongly suspected when the bacterial colony count is ≥10^5^ colony-forming units (CFU) per milliliter (mL) of urine, based on the WHO guidelines (2003), including in asymptomatic girls and women [7]. Currently, the gold standard in establishing a diagnosis of UTI is through the microbiology culture of urine [8]. This culture-based microbiological examination is complex, requires well-trained personnel, takes at least 48 hours to perform, and is cost-ineffective [8]. However, microbiological examination allows the determination of antimicrobial susceptibility, which helps provide appropriate antimicrobial treatments [7].

For acute UTIs, patients often require rapid laboratory results in order to enable immediate management. Hence, a rapid method for urine analysis is examined here. Recently, automated urine flow cytometry has been expected to provide rapid and accurate results in UTI screening [9, 10]. The use of urine flow cytometry with a blue semiconductor laser is expected to provide accurate results in the examination of urine cells and sediment with a fast turnaround time (TAT) of less than 1 minute [11]. This cutting-edge technology can rapidly quantify various parameters including white blood cells (WBCs) and bacteria, which are the key parameters in the diagnosis of UTIs [12]. Nonetheless, standardized microscopic methods are reliable and could be used in setting with limited resources. One of these is the Shih-Yung standardized method [13]. The Shih-Yung method is a reference microscopic method because it uses an insulated tube to ensure that whoever works on the remaining urine sediment retains 0.6 mL. The use of a counting chamber also makes calculations easier and can be converted into high-power fields (HPFs) or microliter (*μ*L) units [13, 14]. In addition, urine chemistry may support UTI screening by providing positive results for leukocyte parameters and often nitrite parameters, whereas the urine sediment of UTI patients provides positive results on leukocyte and bacterial parameters [9, 15].

This study aimed to determine the diagnostic value of the leukocyte and bacterial parameters of automatic urine flow cytometry and calculate the suitability concerning leukocyte and bacterial parameters of flow cytometry versus standardized microscopic methods.

## 2. Materials and Methods

### 2.1. Study Protocol and Samples

**Figure 1:**
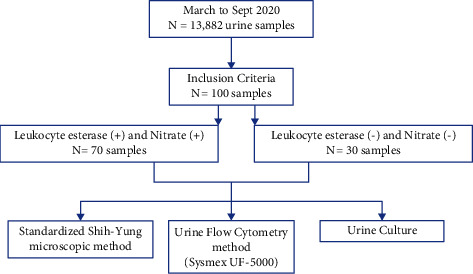
Flow chart of urine specimen analysis.

This was a cross-sectional study, conducted at the Clinical Pathology Laboratory, Dr. Soetomo Hospital, Surabaya, Indonesia. It examined the urinalysis, urine sediment, and urine culture from March 2020 to September 2020. A total of 13,822 urine samples were received during this period at Dr. Soetomo Hospital Clinical Pathology Laboratory. The study aimed to target UTI patients of the outpatient clinic, representing community-acquired UTIs. This group of patients required rapid laboratory results to enable appropriate treatment. For statistical analysis, groups of patients with and without UTIs were established (Figure 1).

All urine samples were processed for 15 different parameters of urine chemistry using a UC-3500 analyzer (Sysmex, Kobe, Japan). Only two parameters were used for inclusion criteria: leukocyte esterase and nitrite. The inclusion criteria for urine samples were first-void midstream urine and being processed within 2 hours after specimen collection; thus, urine specimens processed over 2 hours after the sample collection were excluded. Patients with indwelling catheters were also excluded. Applying these inclusion criteria, a total of 100 urine samples were included for analysis.

The urine samples were then categorized into two groups: the case group and the control group. The case group comprised samples with positive results for leukocyte esterase and nitrite urine (*n* = 70), whereas the control group comprised samples with negative results for both (*n* = 30). The 70 positive samples were classified as patients with suspected UTIs. Based on the treating clinician notes, these 70 patients were confirmed to have UTI symptoms. The 30 negative samples, meanwhile, were classified as the control group, and these 30 were obtained from patients registered for general medical checkups who were also confirmed without UTIs. All urine samples were first-void midstream urine samples, which were obtained from the outpatient unit of Dr. Soetomo Hospital, using a sterile, preservative-free leakproof container (60 mL). The urine analysis was performed within 2 hours after sample collection. To perform this, we made some adjustments to the microscopic quantitation of leukocytes and bacteria based on Strasinger's criteria, which have been adopted in our laboratory to prevent overlapping values. For example, based on Strasinger, the leukocyte urine sediment values are 1–10/HPF and 10–25/HPF, but in our laboratory, we use 1–10/HPF and 11–25/HPF (Table 1) [12].

This study was approved by Dr. Soetomo Hospital Ethical Committee with reference number 038/LOE/301.4.2/III/2021.

### 2.2. Shih-Yung Standardized Microscopic Method

The Shih-Yung method consists of a Shih-Yung quantitative centrifuge tube and a Shih-Yung double-grid counting slide. The urine samples were centrifuged at 400 g for 5 minutes. The tube was separated so that any laboratory personnel on the roster could pour an accurate volume of the supernatant, i.e., 0.6 mL of the urine sediment [13]. WBCs and bacteria were then examined using an HPF microscope.

The Shih-Yung double-grid counting slide provides two types of grids: a Neubauer (conventional) grid and a compressed-area grid. The conventional grid has nine large squares divided into nine small square grids (for a total of 81), whereas the compressed-area grid has four large square grids. Each grid is 1 mm in length, with a chamber depth of 0.05 mm. The large grid is further divided into six small rectangular grids (for a total of 24). If the Neubauer grid was used, a 400x magnification was used. The counting method was diagonally right or left for the nine squares. The total count was then divided by 9 with HPF as the unit. By contrast, if a compressed-area grid was used, the total count of the four squares was divided by 4 and the unit was *μ*L [13, 14].

### 2.3. Sysmex UF-5000

The Sysmex UF-5000 (Sysmex Corporation, Kobe, Japan) is a fully automated urine flow cytometry analyzer with the newest technology, using a blue semiconductor laser at a 488 nm wavelength, with a surface channel (SFch) and a core channel (CRch) analysis chamber [11]. Like its predecessor, the Sysmex UF-5000 analyzer has technology for particle counting and classification based on signals of forward-scattered light (FSC), side-scattered light (SSC), side fluorescent light (SFL), and depolarized side-scattered light (DSS). It determines bacteria using a different light signal for FSC, SFL, and SSC for Gram-negatives and Gram-positives based on different dye intake by cell wall structures. The flagging that appears in the Sysmex UF-5000 analyzer can be BACT: Gram Negative?, BACT: Gram Positive?, and BACT: Gram Pos/Neg? A minimum volume of 2.0 mL of uncentrifuged urine is needed in automated mode and 0.6 mL in the STAT mode. In both the modes, the aspiration volume is 0.45 mL [11].

### 2.4. Microbiological Analysis

The microbiology culture of urine was performed using well-mixed, uncentrifuged samples immediately after they arrived in the laboratory. The urine culture was performed on all samples using a standard 1 *μ*L inoculating loop of a well-mixed urine specimen onto a cystine lactose-deficient agar plate (CLED, Becton Dickinson GmbH, Germany), which supports the growth of Gram-positive and Gram-negative pathogens in urine and prevents the swarming morphology of *Proteus* spp. After inoculation, the Petri dishes were incubated aerobically at 35–37°C for 18–24 hours. The number of colonies was enumerated as CFU/mL. The identification of colonies was performed manually using Gram staining as per the manufacturer's instruction (Sigma-Aldrich, Merck, Germany). The diagnosis of UTI was established when the number of colonies was ≥10^5^ CFU/mL. Microscopic observation from the Gram staining of the bacterial colonies was recorded. A specimen was considered negative in cases of no growth or <10^5^ CFU/mL [7].

### 2.5. Data Analysis

Categorical data from the Sysmex UF-5000 analyzer and the Shih-Yung method were calculated using Cohen's kappa to obtain the agreement between the two methods. To determine the agreement between the two methods, the urine sediment results from automatic urine flow cytometry were categorized for statistical analysis. The categories used are shown in Table 1, with slight modifications to the leukocyte parameters (Table 1). The results of leukocyte and bacterial counts using the Sysmex UF-5000 analyzer were compared with the results of the urine cultures using receiver operating characteristic (ROC) curve analysis.

The interpretation of microscopic quantitation and the dominance angle of the scattergram were blinded. The microscopic interpretation was performed by three well-trained clinical pathologists to minimize subjective interpretation. A minimum of two to three identical quantifications of interpretation were used as confirmed results. When no identical result was obtained by the three pathologists, the interpretation was repeated until two provided the same interpretation. To ensure objective interpretation, no discussion was permitted to take place among the pathologists during the microscopic interpretation.

The flag information for bacteria was included in the data analysis to determine its agreement with the urine culture results.

The data obtained were analyzed using IBM SPSS Statistics 25.0. The results were considered statistically significant if *p* < 0.05.

## 3. Results

Agreement between the Sysmex UF-5000 analyzer and the Shih-Yung method on leukocyte and bacterial parameters was extremely strong (*k* = 0.959, *p* < 0.001, and *k* = 0.939, *p* < 0.001, respectively, Tables 2 and 3). Of the 30 control samples obtained from the medical checkup (MCU) unit and 70-suspected UTI samples, 41 patients (41%) were female. All of the suspected UTI samples showed significant growth, with 63 specimens (90%) recorded as Gram-negative, 1 (1%) as Gram-positive, and 6 (8%) as a mix of Gram-negative and Gram-positive.

**Figure 2:**
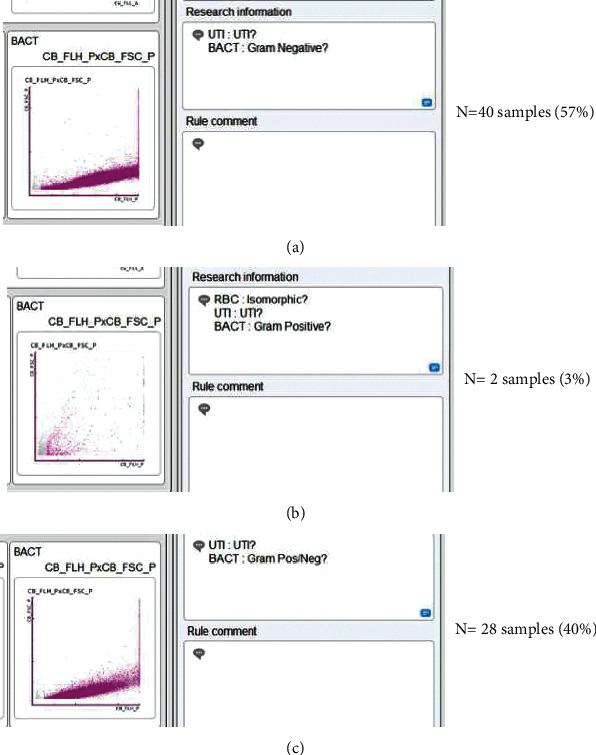
Flagging flow cytometry for bacteria in UTI screening of Gram-negative (a), Gram-positive (b), and mixed Gram-positive and Gram-negative (c).

Forty samples (57%) with suspected UTIs showed flagging “UTI: Gram Negative?,” 28 samples (40%) showed flagging “UTI: Gram Pos/Neg?,” and 2 samples (3%) showed “UTI: Gram Positive?” (Figures 2(a)–2(c)).

**Figure 3:**
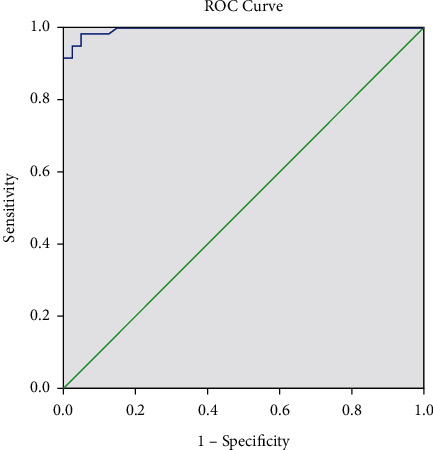
ROC leukocyte parameter.

**Figure 4:**
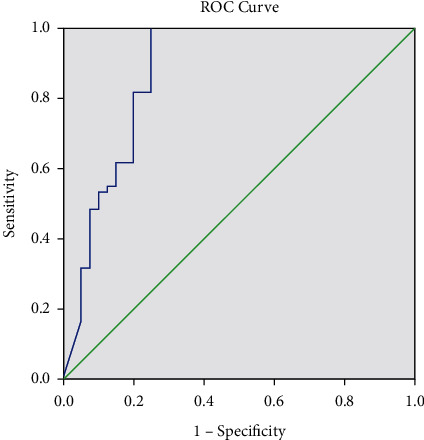
ROC bacteria parameter.

ROC curve analysis performed for the leukocyte parameter indicated an area under the curve (AUC) of 0.995 (*p* < 0.001). At a cutoff of 15.70 leukocytes/HPF equivalent to 87.2/*µL*, the sensitivity (SE) was 98.33%, the specificity (SP) was 95.00%, the positive predictive value (PPV) was 96.72%, and the negative predictive value (NPV) was 97.44% (Figure 3). The ROC curve analysis was also performed for the bacterial parameter and indicated an AUC of 0.873 (*p* < 0.001). At a cutoff of 104.80/HPF equivalent to 582.22/*µL*, the SE was 98.33%, the SP was 75.00%, the PPV was 85.51%, and the NPV was 96.77% (Figure 4).

We also performed analysis on the agreement between the Sysmex UF-5000 flagging and the urine culture results, obtaining a kappa value of 0.212 (*p* < 0.02, Table 4). The fair agreement between bacterial flagging and culture results was due to the presence of mixed flagging Gram-negative and Gram-positive. However, examining only Gram-negative flagging, the result showed good agreement between Gram-negative flagging and the culture (*k* = 0.876, *p* < 0.001).

**Figure 5:**
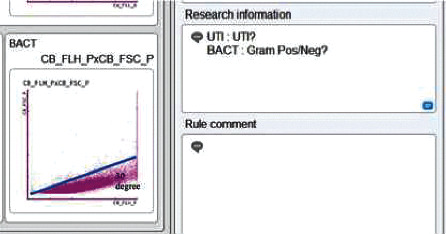
Adjusted dominant angle (<30° was determined as Gram-negative, and ≥30° was determined as Gram-positive).

We then adjusted the data by taking the dominant angle as per the methods described by Asutake et al. and Ozawa et al. [16, 17]. The data with angles <30° and ≥30° were suspected to be Gram-negative and Gram-positive, respectively. The scattergram with the adjustment is illustrated in Figure 5. The result obtained a kappa value of 0.876 (*p* < 0.001, Table 5).

## 4. Discussion

UTIs are among the most common infectious diseases in nonhospitalized and hospitalized patients, and they have a very high mortality rate. They negatively impact the quality of life and cause a burden to the economy due to their high antimicrobial usage [3]. Uropathogenic *Escherichia coli* (UPEC) is the most commonly reported bacteria in community-acquired UTIs, which has led to the increasing use of antimicrobials [3]. The urine culture remains the gold standard in establishing the diagnosis of UTI [8]. However, with the challenges in the requirements for well-trained staff and the inability to provide laboratory results within 24 hours for patients (particularly outpatients), to receive appropriate treatment on time, a rapid approach to providing accurate results through automated urine flow cytometry is highly feasible for the best treatment outcomes.

Indeed, automated urine flow cytometry has become a game-changer in providing a rapid TAT for UTI diagnosis by analyzing the urine sediment. It has been validated by many studies for its reliability, especially in terms of its accuracy [18–22]. Moreover, multiple studies have compared the automated urine flow cytometry approach with microscopic methods and culture results. In this study, however, we incorporated the scattergram dominance in Gram Pos/ Neg? flagging in our data analysis to enable the clinician to provide more accurate antimicrobial treatment decisions based on potential Gram-flagging screening while awaiting the urine microbiological culture results.

Overall, there was extremely strong agreement between the two methods, both for leukocyte and bacterial parameters, with values of *k* = 0.959, *p* < 0.001, and *k* = 0.939, *p* < 0.001, respectively. The Shih-Yung standardized method can be used as a reliable tool for microscopic examination of the urine sediment, especially in remote settings such as primary care laboratories that cannot provide automated urine flow cytometry in areas where the number of examinations is small, but accurate results are required. In line with this study, previous research by Yang et al. found that using Shih-Yung as a microscopic counting method showed good consistency with the urine flow cytometry [19]. In addition, the Shih-Yung method can decrease the subjectivity of volume.

Supernatant removal was performed because it has a separating chamber for the sediment. Having said this, counting cells manually can still be challenging for poorly trained laboratorians.

This study showed reliable and high diagnostic performance for the leukocyte parameter at a cutoff of 87.2/*μ*L, and the bacterial parameter at a cutoff of 582.22/*μ*L. The high NPV in both parameters may improve the screening strategy for UTIs.

Ren et al., meanwhile, analyzed flow cytometry as a reliable tool for discriminating culture-negative urine specimens from patients with suspected UTIs. They showed that good performance of flow cytometry can reduce 61% of unnecessary urine cultures [23]. Furthermore, Gilboe et al. found that using a cutoff value for a bacterial count of ≥100,000/mL and for WBCs of ≥10/*μ*L, flow cytometry predicted 42.1% of samples with nonsignificant growth [24]. The diagnostic performance (SE and SP) has differed across research studies due to the range of methods and cutoffs employed. For instance, De Rosa et al. found other automated urine flow cytometry had an SE of 98.8% and an SP of 76.5% at a cutoff for bacteria of >440/*μ*L and for leukocytes of >150/*μ*L [20], while Gutierrez et al. found that the optimal cutoff values to detect bacteriuria >10^5^ CFU/mL were 690/*μ*L for bacteria and 38/*μ*L for leukocytes, with an SE of 92% and an SP of 65% [21]. Our findings are consistent with previous studies using different types of Sysmex urine flow cytometry and research methods using all urine samples sent to the laboratory. Instead of using all the samples, our study only analyzed samples of suspected community-acquired UTIs with positive nitrite and leukocyte parameters on urinalysis. Thus, our study and those of De Rosa et al. and Gutierrez et al. have provided congruent results of the excellent diagnostic value using automated urine flow cytometry [20, 21].

Asutake et al. showed that most bacterial species with a scattergram low angle pattern (<30°) were Gram-negative bacteria, whereas those that showed that a high angle pattern (≥30°) had both Gram-negative and Gram-positive bacteria in equal measure [16]. Our study conducted a statistical analysis to determine the agreement between the flagging that emerged from the automated urine flow cytometry and the culture results. The flagging that appears can be BACT: Gram-Negative?, BACT: Gram-Positive?, or BACT: Gram-Pos/Neg? The result suggested a moderate level of agreement, with a kappa value of 0.212 and a *p* value of <0.02. Therefore, we conducted further analysis by incorporating the dominance of the angle into the analysis and showed an excellent agreement, with a kappa value of 0.876 and a *p* value of <0.001. The scattergram will appear flagging BACT: Gram-Negative if the angle is <30°. On the other hand, the BACT: the Gram-Positive flag will appear if the angle is ≥30°. Ozawa et al. described the term “wide pattern” as dots distributed over a wide range from low to high angles. This wide pattern showed two or more bacteria identified in 11 of the 14 polymicrobial infections, and *E. coli* was detected in six of the 19 participants who had the wide pattern [17]. From our study, the dominant angle on the scattergram may be a promising predictor of UTI and a basis for providing therapy.

To summarize, this study shows a promising consistent agreement of results using the Shih-Yung microscopic method and the automated urine flow cytometry, indicating that both these methods may be used by clinicians to provide empiric therapy to suspected UTI patients. Thus, this study can be used as a basis for further research with a larger sample size and different instruments.

## 5. Conclusion

Urine flow cytometry and the Shih-Yung method showed excellent agreement. The Shih-Yung method can be used as an accurate method in laboratory settings with few urine sediment examinations, while urine flow cytometry provides accurate results up to the potential Gram-positivity or Gram-negativity of the pathogens with a rapid TAT. Moreover, the urine flow cytometry has a very high NPV and good PPV, SE, and SP for ruling out UTI and giving clinicians a confidence in UTI therapy. Therefore, it has the potential to be used to improve patients' outcomes. Clinicians may use leukocyte and bacterial cutoffs of 87.2/*μ*L and 582.22/*μ*L, respectively, as recommendations to establish the diagnosis of UTI using the flow cytometry method. In addition, the dominant angle of the bacterial scattergram could be used to select antibiotics before urine culture results are received.

## Figures and Tables

**Table 1:** Category of leukocyte and bacterial parameter results.

WBCs/HPF	None: 0 Numerical range: 1–10, 11–25, 26–50, 51–100, >100
BACT/HPF	None: 0 Rare: 1–10 Few: 11–50 Moderate: 51–200 Many: >200

Note: HPF = high-power field.

**Table 2:** Cross-tabulation and a kappa value of Sysmex UF-5000 and Shih-Yung (leukocyte parameter).

WBC (Sysmex UF-5000)	WBC (Shih-Yung)					Total
	>100, *n* (%)	51–100, *n* (%)	26–50, *n* (%)	11–25, *n* (%)	1–10, *n* (%)	
>100	23 (100)	0 (0)	0 (0)	0 (0)	0 (0)	23 (100)
51–100	0 (0)	11 (100)	0 (0)	0 (0)	0 (0)	11 (100)
26–50	0 (0)	0 (0)	10 (76.9)	3 (23.1)	0 (0)	13 (100)
11–25	0 (0)	0 (0)	0 (0)	7 (100)	0 (0)	7 (100)
<10	0 (0)	0 (0)	0 (0)	0 (0)	36 (100)	36 (100)
Kappa	0.959					
*p* value	<0.001					

**Table 3:** Cross-tabulation and a kappa value of Sysmex UF-5000 and Shih-Yung (bacterial parameter).

BACT (Sysmex UF-5000)	BACT (Shih-Yung)					Total
	None, *n* (%)	Rare, *n* (%)	Few, *n* (%)	Moderate, *n* (%)	Many, *n* (%)	
None	1 (100)	0 (0)	0 (0)	0 (0)	0 (0)	1 (100)
Rare	0 (0)	22 (100)	0 (0)	0 (0)	0 (0)	22 (100)
Few	0 (0)	0 (0)	6 (100)	0 (0)	0 (0)	6 (100)
Moderate	0 (0)	0 (0)	0 (0)	2 (66.7)	1(33.3)	3 (100)
Many	0 (0)	0 (0)	0 (0)	2 (2.9)	66 (97.1)	68 (100)
Kappa	0.939					
*p* value	<0.001					

**Table 4:** Agreement between BACT flagging and the urine culture result.

Flagging Sysmex UF-5000	Culture results			Total
	Gram-negative (*n*)	Gram-positive (*n*)	Dual population (*n*)	
Gram-Neg?	38	0	2	40
Gram-Pos?	1	1	0	2
Gram-Neg/Pos?	22	0	6	28
Kappa	0.212			
*p* value	0.002			

**Table 5:** Agreement between angle determination and the urine culture result.

Angle determination Sysmex UF-5000	Culture results			Total
	Gram-negative (*n*)	Gram-positive (*n*)	Dual population (*n*)	
Gram-Neg?	60	0	0	60
Gram-Pos?	0	2	0	2
Gram-Neg/Pos?	2	0	6	8
Kappa	0.876			
*p* value	<0.001			

## Data Availability

The data used to support the findings of this study are available from the corresponding author upon request.
